# Prognostic role of alternative splicing events in head and neck squamous cell carcinoma

**DOI:** 10.1186/s12935-020-01249-0

**Published:** 2020-05-14

**Authors:** Yanni Ding, Guang Feng, Min Yang

**Affiliations:** 1 Department of Breast Surgery, Shaan Xi Provincial Tumor Hospital, Xi’an City, Shaan Xi Province 710000 China; 2 The Third Department of Burns and Plastic Surgery and Center of Wound Repair, The Fourth Medical Center of PLA General Hospital, Beijing, 100048 China

**Keywords:** Head and neck squamous cell carcinoma, Alternative splicing, Splicing factors, Prognosis, The Cancer Genome Atlas

## Abstract

**Background:**

Aberrant alternative splicing (AS) is implicated in biological processes of cancer. This study aims to reveal prognostic AS events and signatures that may serve as prognostic predictors for head and neck squamous cell carcinoma (HNSCC).

**Methods:**

Prognostic AS events in HNSCC were identified by univariate COX analysis. Prognostic signatures comprising prognostic AS events were constructed for prognosis prediction in patients with HNSCC. The correlation between the percent spliced in (PSI) values of AS events and the expression of splicing factors (SFs) was analyzed by Pearson correlation analysis. Gene functional annotation analysis was performed to reveal pathways in which prognostic AS is enriched.

**Results:**

A total of 27,611 AS events in 15,873 genes were observed, and there were 3433 AS events in 2624 genes significantly associated with overall survival (OS) for HNSCC. Moreover, we found that AS prognostic signatures could accurately predict HNSCC prognosis. SF-AS regulatory networks were constructed according to the correlation between PSI values of AS events and the expression levels of SFs.

**Conclusions:**

Our study identified prognostic AS events and signatures. Furthermore, it established SF-AS networks in HNSCC that were valuable in predicting the prognosis of patients with HNSCC and elucidating the regulatory mechanisms underlying AS in HNSCC.

## Background

Recently, substantial progress in the area of high-throughput sequencing technology has motivated cancer genome research. Alternative splicing (AS) is a crucial posttranscriptional biological process that facilitates transcript variants and reprogramming of protein diversity in cells [1]. It is known that dysregulation of AS often causes aberrant cellular homeostasis and is associated with malignancies [2, 3]. Accumulating evidence suggests that AS is implicated in the carcinogenesis and progression of cancers [4, 5]. Furthermore, researchers have found the clinical significance of AS, and it may serve as a prognostic predictor [6]. For instance, Liu et al. reported that that DOCK5 variant promoted proliferation, migration, and invasion of HPV-negative HNSCC cells, and patients with higher expression of DOCK5 variant showed decreased overall survival [7]. A recent study revealed that a novel splice variant of LOXL2 promotes progression of human papillomavirus-negative head and neck squamous cell carcinoma [8]. These studies mainly focus on specific genes, however, studies providing a comprehensive evaluation of splicing events in HNSCC are scarce.

HNSCC is the sixth most common malignancy globally, and remains one of the leading causes of cancer-related death [9]. HNSCC patients require a multimodal approach including surgical resection, radiotherapy, and systemic chemotherapy [10]. However, about half of the patients will develop locoregional recurrence or metastasis, and their five-year survival rates remains unsatisfactory [11]. These facts highlight the urgent need to identify underlying molecular mechanisms to develop effective therapy and improve the overall survival of patients.

RNA sequencing data generated by The Cancer Genome Atlas (TCGA) program enables researchers to illustrate the global profiling of AS events and identify the clinical significance and prognostic value of AS. TCGA SpliceSeq [12] provides valuable processed data for the analysis of AS events in 33 types of cancers, and it includes the following seven types of AS events: alternate acceptor site (AA), alternate donor site (AD), alternate promoter (AP), alternate terminator (AT), exon skip (ES), mutually exclusive exon (ME), and retained intron (RI).

In the present study, we attempted to elucidate the pattern of global aberrant AS events and its clinical and prognostic implications in patients with HNSCC using AS and clinical data obtained from TCGA database. Prognostic AS events that might function as prognostic indicators were identified and AS prognostic signatures were constructed. To assess the regulatory relationships between SFs and AS in HNSCC, a regulatory network was also established.

## Materials and methods

### TCGA data process

TCGA SpliceSeq is a database that provides AS profiles for seven types of splice events across 33 tumors based on TCGA RNA-seq data. To quantify AS events, the percent spliced in (PSI) value was processed for further data analysis. The PSI value indicates the inclusion of a transcript element divided by the total number of reads for that AS event. Alterations in PSI values range from 0 to 100%, which suggests a shift percentage in splicing events. AS events with a PSI value of more than 75% in a HNSCC cohort were obtained from TCGA SpliceSeq website. AS events with a standard diversion < 1 were excluded. An UpSet plot generated by the package “UpSetR” [13] in R software was used for analyzing and displaying the distribution and intersection among seven types of AS. Clinical information of patients with HNSCC was also downloaded and extracted from TCGA database. The overall survival (OS) was used as the endpoint for survival.

### Survival analysis of AS events

In the survival analysis, the follow-up periods ranged from 91 to 6417 days after excluding patients with an OS of less than 90 days. Univariate Cox analysis was conducted to assess the correlation between the survival status of patients with HNSCC and PSI value (from 0 to 100) of each AS event (P < 0.05). A total of 486 patients with HNSCC were ranked from low to high according to risk score, and the median were used as cut-off to separate patients into low- or high- risk groups. Therefore, there are 243 patients in each group.

### Prognostic signature construction

The top 20 most significant events of seven types of AS from the univariate Cox analysis were submitted to a least absolute shrinkage and selection operator (LASSO) analysis to develop prognostic signatures. The coefficients and partial likelihood deviance were calculated with the “glmnet” [14] package in R. The prognostic signatures for OS prediction were calculated by multiplying the PSI values of prognostic indicators with the coefficients assigned by LASSO Cox analysis. Evaluation of the splicing-based prognostic signature as an independent predictor was conducted by integrating the following clinical parameters into the multivariable Cox regression analysis: age, gender, tumor stage, lymph node status, distant metastasis, tumor-node-metastasis (TNM) stage and histological grade. The prognostic prediction efficacy of the AS signatures was examined by a time-dependent receiver-operator characteristic (ROC) curve and analyzed using the package “survivalROC” [15] in R software. The risk score calculated by the package “pheatmap” [16] was to evaluate the performance of the prognostic signatures. The Kaplan–Meier survival analysis was conducted to assess the survival difference between high- and low-risk groups.

### SF-AS regulatory network

A list of 404 splicing factors was obtained from the database of SpliceAid 2 [17]. The expression profiles of SF genes were selected from TCGA dataset. Correlation between the PSI values of prognostic AS events and the expression of SFs was examined by Pearson’s correlation analysis. SF-AS relationships with P value less than 0.05 and a Pearson correlation coefficient more than 0.65 were selected to construct the SF-AS a regulatory network via Cytoscape version 3.6.1.

### Functional annotation

Functional annotation of genes with prognostic AS events was performed by the package “clusterProfiler” [43] in R to investigate the functional relevance of the genes involved in AS events. Kyoto Encyclopedia of Genes and Genomes (KEGG) and the Gene ontology (GO) were used to assess the functional categories. KEGG and GO terms with a P-value and q-value both smaller than 0.05 were considered significant categories.

## Results

### Profiles of alternative splicing events in HNSCC

**Fig. 1:**
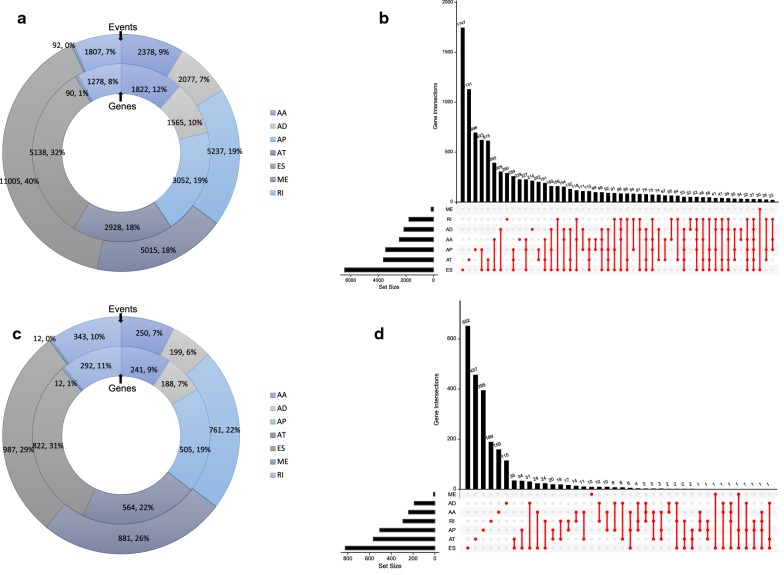
Overview of alternative splicing (AS) events and prognostic AS events in HNSCC. **a** Numbers and percentages of events and corresponding genes in seven types of AS; **b** UpSet plots showing the intersection of seven types of AS events; **c** numbers and percentages of prognosis- associated AS events and corresponding genes; **d** UpSet plots showing the intersection of prognostic AS events

We processed TCGA splice-seq data and clinical information of a HNSCC cohort in TCGA, and a total of 347 patients were included in the analysis. In total, 27,611 AS events in 15,873 mRNAs were observed in HNSCC, indicating that AS events are common in the development of HNSCC. The specific numbers and percentages of events and corresponding genes in seven types of AS were showed in Fig. 1a. An UpSet graph was generated to analyze the intersection among seven types of AS and to display the distribution of spliced genes in different splicing types (Fig. 1b). We found that one gene may have multiple types of splicing events, and that ES was the most predominant type.

### Prognostic AS events

To reveal the prognostic significance of AS events in patients with HNSCC, a univariate Cox analysis on AS events was performed. The specific numbers and percentages of prognosis-associated AS events and corresponding genes were showed in Fig. 1c. Additionally, one gene could present two or more AS events that were markedly related to the OS of patients with HNSCC. The UpSet plot demonstrated that ES was still the leading prognostic AS type, and that a gene could have up to three prognostic events (Fig. 1d).

### Prognostic signatures for patients with HNSCC

**Fig. 2:**
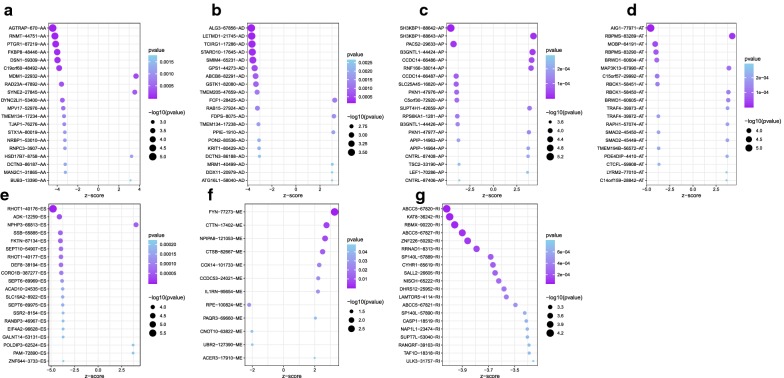
The top 20 most significant AS events in HNSCC. **a** alternate acceptor, **b** alternate donor sites, **c** alternate promoters, **d** alternate terminators, **e** exon skips, **f** mutually exclusive exons, and **g** retained introns

**Fig. 3:**
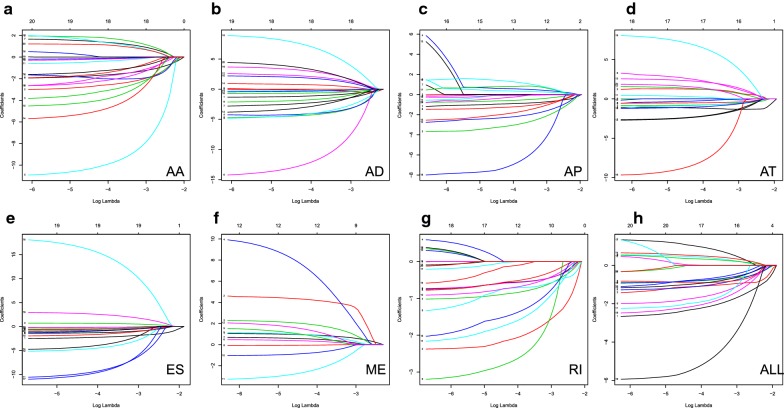
Construction of prognostic signatures based on LASSO COX analysis. **a** alternate acceptor, **b** alternate donor sites, **c** alternate promoters, **d** alternate terminators, **e** exon skips, **f** mutually exclusive exons, **g** retained introns, and **h** comprehensive signature

**Fig. 4:**
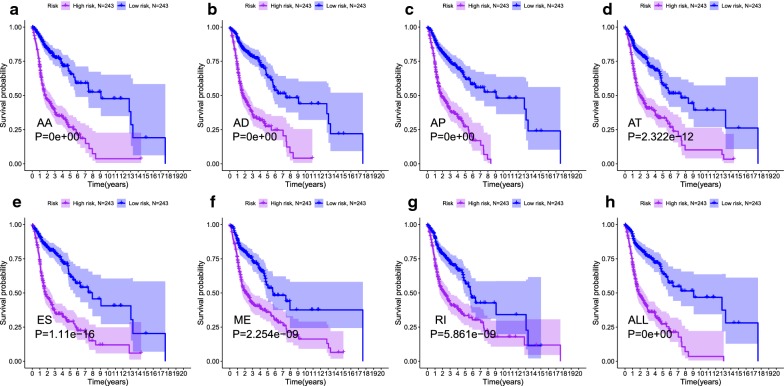
Kaplan-Meier curves of high risk (red) and low risk (blue) HNSCC patients according to eight prognostic signatures. **a** alternate acceptor, **b** alternate donor sites, **c** alternate promoters, **d** alternate terminators, **e** exon skips, **f** mutually exclusive exons, **g** retained introns, and **h** comprehensive signature

**Fig. 5:**
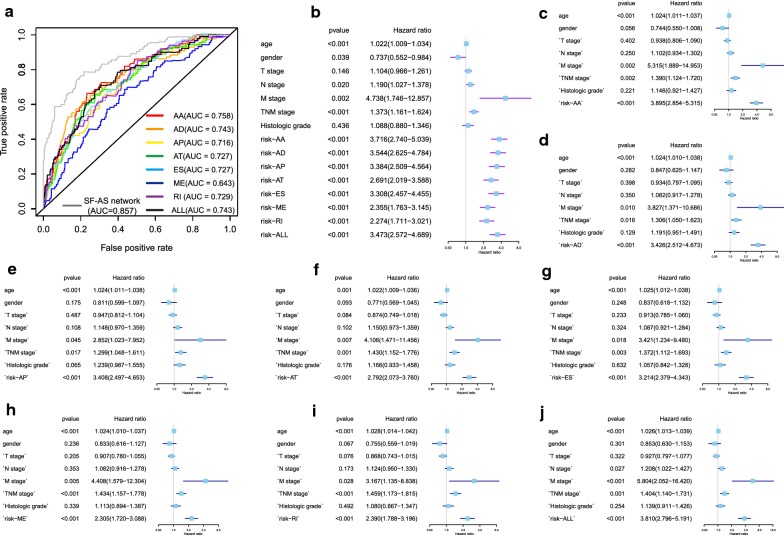
**a** ROC curves of prognostic signatures for HNSCC. **b** Univariate Cox regression analysis of clinical features and prognostic signatures. **c**–**j** Multivariate analysis of clinicopathological features and eight prognostic signatures. **c** alternate acceptor, **d** alternate donor sites, **e** alternate promoters, **f** alternate terminators, **g** exon skips, **h** mutually exclusive exons, **i** retained introns, and **j** comprehensive signature

**Fig. 6:**
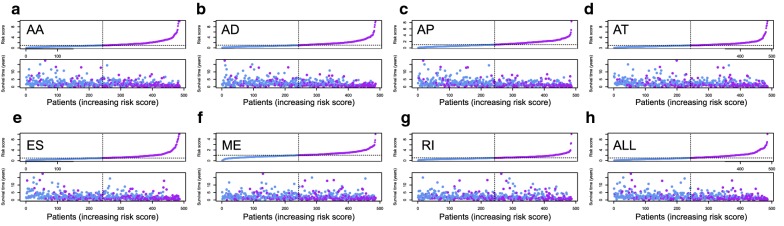
The risk scores and distribution of survival time of eight signatures in patients with HNSCC. **a** alternate acceptor, **b** alternate donor sites, **c** alternate promoters, **d** alternate terminators, **e** exon skips, **f** mutually exclusive exons, **g** retained introns, and **h** comprehensive signature. Upper plot: risk score; Lower plot: survival time distribution

The 20 most significant prognostic events of each of the seven AS types are shown in Fig. 2a–g. By using the LASSO Cox analysis, we developed seven types of prognostic signatures based on prognostic AA, AD, AP, AT, ES, ME, and RI events (Fig. 3a–g). Additionally, we conducted an integrated analysis of all the seven types of AS events to establish a comprehensive prognostic signature (abbreviated as “ALL”), which consist of RHOT1-40176-ES, SH3KBP1-88642-AP, AGTRAP-670-AA, SH3KBP1-88643-AP, PACS2-29633-AP, RBPMS-83289-AT, B3GNTL1-44424-AP, MOBP-64191-AT, NPHP3-66813-ES, ABCC5-67820-RI, FKTN-87134-ES and FKBP8-48446-AA (Fig. 3h). The detailed information of these eight prognostic signatures is listed in Table 1. As expected, Kaplan–Meier analysis suggested that the seven prognostic signatures could effectively separate the survival curves of high-risk groups from those of the low-risk groups (Fig. 4a–g), and the comprehensive prognostic signature could accurately predict prognosis (Fig. 4h). ROC curves further validated the efficacy of these eight prognostic signatures in prognosis prediction, and the area under the cure (AUC) of eight signatures was larger than 0.7, and the AUC of comprehensive signatures is 0.743 (Fig. 5a). We next performed univariate Cox regression analysis and found that the eight signatures had a high predictive value regarding the OS of patients with HNSCC (Fig. 5b). Furthermore, all eight signatures remained independent prognostic indicators for patients with HNSCC in multivariate analyses after other clinicopathological characteristics were adjusted (Fig. 5c–j). The risk scores of eight signatures in patients with HNSCC were ranked from low to high, as shown in Fig. 6a–h, and the median was used as a cut-off to divide high- and low-risk groups (upper panel). Patients with a high-risk score tended to have lower survival rate and shorter survival time (lower panel).

**Table 1:** Alternative splicing signatures associated with overall survival in patients with HNSC

AS type	Formula	HR (95% CI)	AUC
AA	(AGTRAP|670|AA × − 2.12)+(RNMT|44751|AA × − 3.18)+(PTGR1|87219|AA × − 4.27)+(DSN1|59309|AA × − 10.59)+(C19orf60|48492|AA × − 2.08)+(MDM1|22932|AA × 1.56)+(RAD23A|47892|AA × − 2.79)+(SYNE2|27845|AA × 2.23)+(TMEM134|17234|AA × − 2.65)+(TJAP1|76276|AA × − 1.27)+(STX1A|80019|AA × − 5.92)+(NRBP1|53010|AA × − 4.98)+(HSD17B7|8758|AA × 1.98)+(BUB3|13390|AA × 1.63)	3.716 (2.740–5.039)	0.758
AD	(ALG3|67856|AD × − 1.51)+(TCIRG1|17286|AD × − 4.93)+(SMIM4|65231|AD × − 5.2)+(GPS1|44273|AD × − 14.83)+(ABCB8|82291|AD × − 3.26)+(TMEM205|47659|AD × − 2.3)+(FCF1|28425|AD × 2.31)+(FDPS|8075|AD × 2.5)+(TMEM134|17238|AD × − 4.29)+(KRIT1|80429|AD × − 0.78)+(DCTN3|86188|AD × − 0.48)+(MRM1|40499|AD × 9.4)+(DDX11|20979|AD × 3.86)+(ATG16L1|58040|AD × 4.86)	3.544 (2.625–4.784)	0.743
AP	(SH3KBP1|88642|AP × − 1.08)+(PACS2|29633|AP × − 3.7)+(B3GNTL1|44424|AP × 0.74)+(SLC25A45|16820|AP × − 1.41)+(PKN1|47976|AP × − 0.75)+(C5orf30|72920|AP × − 2.54)+(APIP|14963|AP × − 2.68)+(TSC2|33190|AP × − 8.59)+(LEF1|70286|AP × 1.73)+(CNTRL|87406|AP × − 1.32)	3.384 (2.509–4.564)	0.716
AT	(RBPMS|83289|AT × 2.65)+(MOBP|64191|AT × − 0.87)+(C15orf57|29992|AT × − 3.25)+(RAPH1|57074|AT × 2.73)+(SMAD2|45450|AT × − 2.81)+(TMEM194B|56572|AT × − 10.03)+(PDE4DIP|4410|AT × 1.73)+(CTCFL|59908|AT × − 1.3)+(LYRM2|77010|AT × 11.22)+(C14orf159|28842|AT × 2.38)	2.691 (2.019–3.588)	0.727
ES	(RHOT1|40176|ES × − 3.19)+(NPHP3|66813|ES × 0.96)+(FKTN|87134|ES × − 1.61)+(DEF8|38194|ES × − 2.08)+(CORO1B|387277|ES × − 0.65)+(ACAD10|24535|ES × − 11.34)+(SLC19A2|8922|ES × − 5.85)+(SSR2|8154|ES × − 4.18)+(RANBP3|46967|ES × − 2.17)+(GALNT14|53131|ES × − 11.11)+(POLDIP3|62524|ES × 19.11)+(PAM|72890|ES × 3.45)	3.308 (2.457–4.455)	0.727
ME	(FYN|77273|ME × 1.19)+(NPIPA8|121053|ME × 2.9)+(CTSB|82667|ME × 10.62)+(COX14|101733|ME × 1.2)+(IL1RN|95654|ME × 0.82)+(CNOT10|63822|ME × − 2.44)+(UBR2|127390|ME × − 3.37)	2.355 (1.763–3.145)	0.643
RI	(ABCC5|67820|RI × − 0.71)+(KAT8|36242|RI × − 2.81)+(RBMX|90220|RI × − 1.03)+(CYHR1|85619|RI × − 0.87)+(SALL2|26605|RI × − 3.1)+(DHRS12|25952|RI × − 2.38)+(LAMTOR5|4114|RI × − 0.93)+(NAP1L1|23474|RI × − 1.73)	2.274 (1.711–3.021)	0.729
ALL	(RHOT1|40176|ES × − 2.85)+(SH3KBP1|88642|AP × − 1.07)+(AGTRAP|670|AA × − 1.28)+(SH3KBP1|88643|AP × 0.59)+(PACS2|29633|AP × − 2.14)+(RBPMS|83289|AT × 2.17)+(B3GNTL1|44424|AP × 0.68)+(MOBP|64191|AT × − 1.18)+(NPHP3|66813|ES × 0.67)+(ABCC5|67820|RI × − 1.01)+(FKTN|87134|ES × − 2.54)+(FKBP8|48446|AA × − 6.71)	3.473 (2.572–4.689)	0.743

AS, alternative splicing; HR, hazard ratio; AUC, area under curve; AA, alternate acceptor; AD, alternate donor sites; AP, alternate promoters; AT, alternate terminators; ES, exon skips, ME, mutually exclusive exons; RI, retained introns; ALL, all types

### Prognostic SF-AS network

**Fig. 7:**
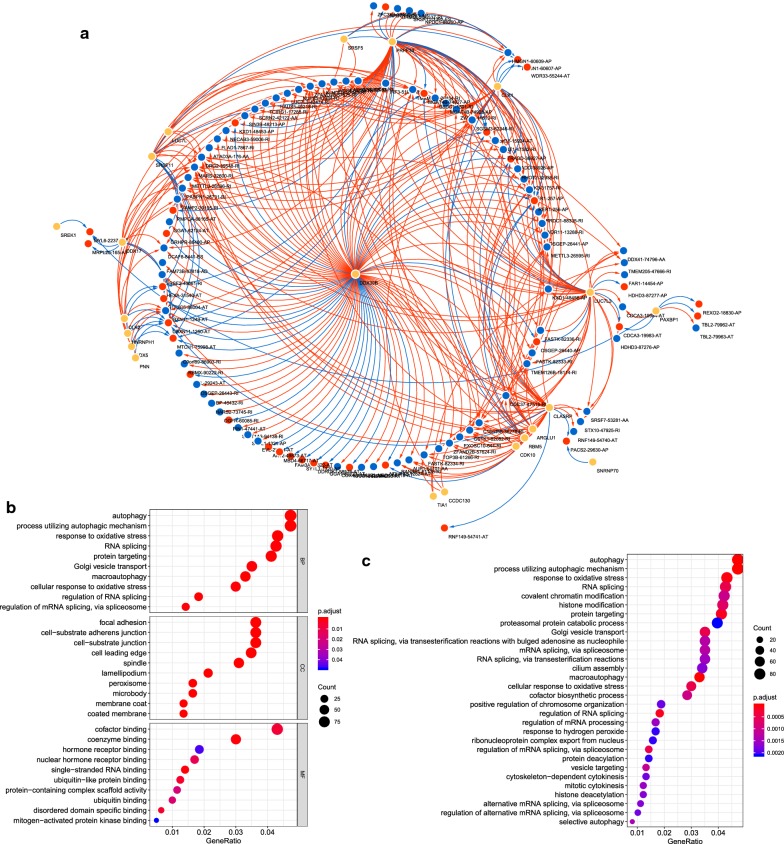
**a** Prognostic SF-AS network in HNSCC. Red/blue line represents positively/negative correlation; red/blue ellipse represents risk/protective AS events; yellow ellipse represents splicing factors. **b**–**c** Bubble plot displayed the GO (**b**) and KEGG (**c**) analysis of genes with prognostic alternative splicing events

It is known that AS events are influenced by SFs [18]. Hence, exploration of the SF-AS regulatory network is important to reveal the mechanism underlying AS in HNSCC. The Pearson correlation analysis indicated that there were 23 splicing factors positively correlated with 84 AS events, whereas 22 splicing factors were negatively correlated with 37 AS events. An interaction network was constructed according to the correlation between SF and AS, which comprises 28 SFs, 33 risk AS events (associated with poor prognosis), and 78 protective AS events (associated with good prognosis) (Fig. 7a). Among the network, splicing factors DDX39B, PRPF39, LUC7L3 and CLASRP were significantly correlated with more than 30 AS events. Notably, DDX39B directly regulates 86 AS events, therefore it was considered as a core SF.

### Functional enrichment analysis

Functional enrichment analysis including the KEGG pathways enrichment and GO analysis were performed for survival‐related AS genes. The results of GO analysis indicated that AS genes were involved in biological processes such as autophagy and RNA splicing (Fig. 7b). In the KEGG analysis, genes corresponding to the prognosis-associated AS events were mainly enriched in cancer-related pathways, for instance, “autophagy”, “response to oxidative stress”, and “RNA splicing” (Fig. 7c).

## Discussion

AS is responsible for the modification of mRNA isoforms, and it plays an indispensable role in producing various mRNA and protein isoforms with multiple functions [19]. Accumulating evidence has revealed that aberrant AS is implicated in the oncogenic processes of multiple malignancies [20, 21]. Therefore, investigation of AS mechanisms deepens our understanding of posttranscriptional regulatory patterns.

In recent years, next-generation sequencing technology has extensively promoted research at a whole-genome scale. RNA sequencing data from TCGA database have enabled the studies of AS patterns in various cancer types [22–24]. By using the SpliceSeq database, several studies explored alternative splicing profiles and constructed prognostic signatures for many types of cancers, including colorectal cancer [22], prostate adenocarcinoma [25], esophageal carcinoma [26], hepatocellular carcinoma [27, 28], kidney renal clear cell carcinoma [29], and soft tissue sarcoma [30]. However, a comprehensive study regarding aberrant AS events in HNSCC is deficient. Li et al. carried out a systemic bioinformatic analysis on the genome-wide AS events of clinical HNSCC samples from the TCGA database [31]. However, this study failed to identify AS events as independent prognostic predictors, and the authors did not explore the interaction between SFs and AS events. Another genome-wide analysis of the AS landscape in HNSC revealed novel AS events related to carcinogenesis and immune microenvironment, with implications for prognosis and therapeutic responses [32]. This study revealed role of each individual AS events and genes in HNSCC immune microenvironment, instead of constructing of comprehensive AS prognostic signature or regulatory network. In the present study, a total of 27,611 AS events in 15,873 mRNAs were observed in HNSCC, indicating that AS events are common in the development of HNSCC. Results of the survival analysis suggest that 3433 AS events in 2624 genes are associated with the OS of patients with HNSCC. We constructed seven splicing prognostic signatures based on seven types of prognostic AS events. Additionally, a comprehensive prognostic signature was developed by integrating all seven types of AS. Genes and AS events enrolled in the comprehensive prognostic signature included RHOT1 (ES) [33], SH3KBP1 (AP) [34], AGTRAP (AA) [35], PACS2 (AP) [36], RBPMS (AT) [37], B3GNTL1 (AP) [38], MOBP (AT), NPHP3 (ES), ABCC5 (RI) [39], FKTN (ES) and FKBP8 (AA) [40], many of which are known to play important roles in cancer biology. For instance, RHOT1 can regulate cell migration and proliferation by suppressing the expression of SMAD4 in pancreatic cancer [33]. PACS-2 as a phosphorylation-state dependent molecular switch that mediates either antiapoptotic or pro-apoptotic signaling [36]. Mourskaia et al. suggest that ABCC5 functions as a mediator of breast cancer skeletal metastasis [39]. FKBP8 is an endogenous inhibitor of mTOR, its degradation promotes tumor progression [40].

The AUC value of the comprehensive signature has reached 0.743, indicating that the prognostic biomarkers can be a useful tool to predict the prognosis of patients with HNSCC. These signatures could accurately distinguish between patients with HNSCC with distinct clinical outcomes, which confirmed that these prognostic signatures could serve as ideal predictors. Moreover, an SF-AS network was constructed to provide further insights into the regulatory mechanisms underlying AS in HNSCC. We found that DDX39B might act as core SFs because of their extensive correlation with AS events. Awasthi et al. found that increase in DDX39B enhances global translation and cell proliferation through upregulation of pre-ribosomal RNA, and dysregulation of DDX39B expression could lead to oncogenesis [41]. Nakata identified DDX39 as a potential drug target for the treatment of AR splice variant-positive prostate cancer. The authors reported that DDX39B and its paralog DDX39A regulated androgen receptor splice variant AR-V7 generation [42].

HPV infection status is a critical factor in the carcinogenesis and progression of HNSCC. Therefore, we check the HPV status in the clinical data of TCGA-HNSC dataset as the reviewer suggested, and found that only 89 out of 528 patients were tested for HPV status (negative = 67, positive = 22). Subsequently, HPV positive and negative samples were integrated to identify differentially expressed alternative splicing. However, we failed to find significantly different AS events between HPV positive and negative samples with the threshold of |log2FC| > 1 and adj P Value < 0.05 (t-test). We think this is because the data of HPV status in most patients is absent, the differential analysis cannot provide accurate results.

## Conclusion

We identified prognostic AS events based on the data from TCGA database. The constructed prognostic AS signatures could effectively predict survival outcomes of patients with HNSCC. The constructed prognostic SF-AS regulatory network may reveal the mechanisms underlying AS in the carcinogenesis of HNSCC. This in-depth analysis of AS in HNSCC may provide useful tools for prognosis prediction and clues for possible therapeutic targets for future clinical applications.

## Data Availability

Not applicable.
